# New insights into an old question: can the MD-PhD physician-scientist pipeline be improved?

**DOI:** 10.1172/jci.insight.158466

**Published:** 2022-03-22

**Authors:** Ronald J. Koenig

**Affiliations:** Division of Metabolism, Endocrinology and Diabetes, University of Michigan Medical School, Ann Arbor, Michigan, USA.

The US needs more physician-scientists. This was a major conclusion of the NIH-sponsored 2014 Physician-Scientist Workforce Report (1). As noted in that report, physician-scientists constitute only 1.5% of the total physician workforce. This percentage has been decreasing, and the average age of physician-scientists has been increasing, which leads to the conclusion that the current physician-scientist pipeline is inadequate to meet our country’s future needs. MD-PhDs are a critical component of this pipeline. They constitute about 50% of the physician-scientist workforce and are uniquely trained to integrate the complexities of basic biomedical science with those of clinical medicine.

In 1964, the National Institute of General Medical Sciences (NIGMS) established Medical Scientist Training Program (MSTP) institutional T32 grants to foster and support combined MD-PhD degree education. Currently, there are approximately 50 NIGMS-funded MSTPs and nearly as many non-NIH-funded MD-PhD programs, which together awarded 620 combined MD-PhD degrees in 2020 (20,387 MD degrees were awarded by US medical schools in 2020) (2).

It is not difficult to think of reasons why the MD-PhD pipeline is so small. Commonly cited reasons include, among others, the length of training and its associated opportunity costs, and the difficulty of sustaining research funding over the course of a career. Data from the American Association of Medical Colleges (AAMC) National MD-PhD program outcomes study (3, 4) showed that the average time to degree for MD-PhD students increased from 6.69 years for the cohort that graduated between 1975 and 1984 to 8.25 years for the cohort that graduated between 2005 and 2014. Postgraduate training also has lengthened. The average age of MD-PhDs with NIH Research Project Grants (RPGs) was 48 years in 2003 and 52 years in 2012; the average age of *first-time* MD-PhD RPG holders was 44.3 years in 2012.

Two recent articles in *JCI Insight* address these MD-PhD pipeline concerns: Brass and colleagues investigated the front end of the pipeline (prior to submitting MD-PhD program applications) (5), whereas Ghosh-Choudhary and colleagues investigated the back end (after graduation) (6). Both provide important insights and much food for thought, while raising numerous unanswered questions.

Brass and colleagues investigated an aspect of the aging MD-PhD workforce that has not previously been studied in-depth: the increasing tendency of students to take gap years after graduating from college and before enrolling in an MD-PhD program (5). In January 2021, the authors invited the directors of US MD-PhD programs and their students to participate in a survey to study students’ reasons for taking a gap between college and MD-PhD program matriculation. Seventy-three MD-PhD programs, including 49 of the 50 NIGMS-supported MSTPs, agreed to participate. Completed surveys were received from 71% of students (3544 of 5007) enrolled in those programs, and the data were supplemented by deidentified information obtained from the AAMC. Seventy-two of the 73 directors of the participating programs also provided data. Forty-one directors answered a subsequent request for deidentified information on program graduates who matriculated in 2006 or later. This study has numerous strengths, including the relatively impressive survey response rates, the comprehensive and well thought out questions, and the analytical methodology.

Several highlights of the findings are noted here. The percentage of MD-PhD students who have taken at least one gap year after college has increased from 53% of those who entered in 2013 to 75% of those who entered in 2020 (5). The primary reason for taking a gap year(s) was to do more research, either to make the applicant more competitive or to help decide whether to apply, and 75% of those who took a gap answered “yes” to the question “Did you feel that a gap year was necessary to maximize your candidacy?” Not surprisingly, students with less undergraduate research experience were more likely to take a gap, and students with social science majors were more likely to do so than students with biological or physical science majors. Although gap years also have become more common among MD students, the magnitude of change has been more modest (from ~57% in 2013 to ~65% in 2020) and the reasons for the gap years differ, often including working in a different career or working to improve finances.

In the program directors’ survey, 51 of 70 directors (73%) said they take gaps into consideration for interview and admission decisions, whereas 19 directors (27%) said they did not (5). Only 5 directors (7%) said they strongly prefer that applicants have taken a gap. The authors conclude that, on average, applicants seem to think a gap is more important than program directors do. However, program directors might be underestimating the weight they place on gap years, which likely result in more sophisticated and compelling application essays and stronger letters of recommendation, etc., compared with what that same applicant would have presented without a gap year, thereby influencing that applicant’s admissions outcome. Interestingly, 1 or 2 gap years had no effect on time to degree (the average time to dual degree was 8.2 years).

The increasing prevalence of gap years may largely be due to a snowball effect: as more MD-PhD students take gap years, potential applicants conclude that doing so will enhance their chances of admission. This conclusion gets reenforced by prehealth advisors and MD-PhD program directors, who indicate that more research experience is better before applying, even if many of those directors responded in the survey that gap years were not strongly preferred.

An important point is that NIH expectations may be contributing to the increasing prevalence of gap years. NIGMS MSTP T32 applications require detailed data on the number of months of prior research experience that applicants and matriculants have. The natural consequence is that program directors will conclude more is better, and their admissions decisions will reflect this.

The authors speculate that the main potential harm related to gap years becoming so common is that it may dissuade individuals from applying, especially on top of the already-long duration of training and the associated opportunity costs (5). This may be particularly significant for underrepresented groups and economically disadvantaged individuals, which is of particular concern given the suboptimal level of diversity among MD-PhDs. Furthermore, the authors point out that there are no data to indicate that gap years improve short- or long-term outcomes. The authors “suggest that policies and practices be revisited to assess their effect on applicants and societal needs for an active and diverse physician-scientist workforce” (5). This is a desirable goal, but it is difficult to know what would constitute a better approach. After all, the MD-PhD application process is extremely competitive, and if one were to downplay the value of gap years, what would replace it? Furthermore, research-focused gap years may enhance MD-PhD program diversity by giving applicants from small schools with little research infrastructure (which include a substantial number of economically disadvantaged individuals and those from underrepresented groups) a chance to catch up in their research experience, obtain stronger letters of recommendation, and better assess whether an MD-PhD is right for them. These advantages of gap years may improve the outcomes of some students and may appropriately dissuade others from applying, although this survey is not able to test such hypotheses. Survey data generally are not well suited to investigate causality, and therefore currently it would be quite challenging to predict whether any specific alteration in MD-PhD admissions policy would be of net societal benefit.

It is interesting to note that the increasing prevalence of gap years among MD-PD applicants and the increasing age at which graduates enter the job market appear to reflect a broader generational change in which career and family decisions are delayed. For example, the percentage of entering University of Michigan Law School students who have taken a gap year increased from 64% in 2003 to 80% in 2018 (personal communication, Law School Admissions Office). The average age of first-time mothers increased from 21 in 1972 to 26 in 2018 (the average age of first-time mothers with a college degree is 30). The average age of first-time fathers increased from 27 in 1972 to 31 in 2018 (7).

Ghosh-Choudhary and colleagues investigated the funding trajectories of MD-PhD students during their training and beyond (6). Due to longstanding concerns about the inadequacy of the pipeline, the NIH has established several mechanisms to help support and foster the success of physician-scientists. One such mechanism, the F30 National Research Service Award (NRSA), was established in 1990 to support individual dual-degree (mostly MD-PhD) students during their graduate training. The “classic” pathway to independence for dual-degree physician-scientist has been to obtain subsequently an NIH K award during postgraduate training (e.g., K08 Mentored Clinical Scientist Research Career Development Award) and then an R award when fully independent (e.g., R01 RPG).

Using the publicly available NIH RePORTER database (https://reporter.nih.gov/), the authors found that 16.7% of male and 14.1% of female MD-PhD students who received an F30 award between 1990 and 2012 subsequently received a K award, and this gender difference was not significant (*P* = 0.30) (6).

To study the F30-to-R transition, the authors restricted the F30 funding period to 1990–2007, due to the longer time frame needed for this transition. In contrast to F30 and K awards, R awards were heavily skewed toward men. Twenty-four percent of male F30 awardees, but only 9.3% of female awardees, received an R award (*P* = 0.001). Obtaining a K award did not overcome this gender disparity, as the K-to-R transition rate was 56% for men and 29% for women. Of interest, receiving a K award did not shorten the time from F30 to R award.

These data raise several areas of concern: the relatively small fraction of F30 awardees who go on to receive a K award, the even smaller fraction who receive an R grant, and especially, the small fraction of women who receive R funding. As the authors note, similarly low rates have been reported previously (8–12).

Based on the low F30-to-R transition rate, one might question whether F30 grants help train physician-scientists. However, this study did not compare F30 recipients to non-recipients (6). It should be noted that not all NIH institutes participate in the F30 mechanism, and those that do have changed over time. In addition, the number of male and female MD-PhD F30 recipients who applied for K and R awards is unknown, which makes it impossible to know whether the low F30-to-K or -R transition rates are due to very few MD-PhDs applying or to very low success rates of submitted applications. These limitations make it difficult to assess questions such as the following. Are F30 and K grants good training experiences? Do the low rates of K and R funding reflect deficiencies in training within MD-PhD programs or issues that occur after graduation? What should be done to mitigate these low transition rates?

The long time frame inherent to the F30-to-R transition is also somewhat of a confounding factor. Between 1990 and 2007, there were 433 F30 awards, but the NIH subsequently ramped up F30 funding such that inspection of Figure 2 of Ghosh-Choudhary and colleagues (6) indicates that approximately 8 times as many F30 awards per year were made in 2013–2019 compared with the previous time frame. The small size of the 1990–2007 cohort and the long time frame from F30 to R award lead to uncertainty as to whether the experiences of the 1990–2007 F30 awardees will be predictive of those of more recent recipients.

Despite the above limitations, the authors present an excellent discussion of potential ways to mitigate the low F30-to-R transition rate and an especially thorough discussion of factors that might underlie the very low transition rate for women. The authors make the important point that R-level funding is an inadequate measure of physician-scientist success, given the many career paths that MD-PhDs take and the important contributions they make to research that are funded through non-NIH sources. A better, more inclusive, measure of success needs to be developed. Overall, these two papers provide interesting, important insights into the MD-PhD pipeline problem and raise numerous questions that should be addressed in future studies.

## Version 1 03/22/2022

Electronic publication
